# Analytical dataset of short-term heat stress induced reshuffling of metabolism and transcriptomes in maize grown under elevated CO_2_

**DOI:** 10.1016/j.dib.2019.105004

**Published:** 2019-12-17

**Authors:** Jemaa Essemine, Jikai Li, Genyun Chen, Mingnan Qu

**Affiliations:** a CAS Center for Excellence in Molecular Plant Sciences, Institute of Plant Physiology and Ecology, Shanghai Institutes for Biological Sciences, Chinese Academy of Sciences, 200032, Shanghai, China; b Institute of Grass Research, Heilongjiang Academy of Agricultural Sciences, Harbin, China

**Keywords:** Metabolism, Transcriptomes, Sudden heat stress, Elevated CO_2_, Maize

## Abstract

This data article describes the analysis of sudden heat stress (SHS) induced transcriptomes and metabolism in SQ maize cultivar (*Zea mays* L. cv. Silver Queen). Plants were grown under elevated CO_2_ in both field based open top chambers (OTCs) and indoor growth chamber conditions [1]. After 20 days after radicle emergence, intact leaf section of maize was exposed for 2 hours to SHS treatment. Samples were stored in liquid nitrogen immediately and used thereafter for metabolism and transcriptomes determinations. Metabolism consisting of 37 targeted metabolites together with corresponding reference standard were determined by gas chromatography coupled to mass spectrometry (GC-MS). Total RNA was extracted using TRIzol® reagent according to the manufacturer's instructions (Invitrogen, Carlsbad, CA). RNA integrity was assessed using RNA Nano 6000 Assay Kit of the Agilent Bioanalyzer 2100 system (Agilent Technologies, CA, USA). Transcriptomes were determined by Illumina Hiseq 4000 platform. Further interpretation and discussion on these datasets can be found in the related article entitled “Elevated CO_2_ concentrations may alleviate the detrimental effects of sudden heat stress on photosynthetic carbon metabolism in maize” [1].

**Table:** Specifications Table

Subject	Agricultural and Biological Sciences (General)
Specific subject area	Heat stress induced modulation in metabolism and transcriptomes in maize
Type of data	Tables (Microsoft word) Figures (TIFF format)
How data were acquired	GC-MS: gas chromatography coupled to mass spectrometry (GC-MS; 7890 GC system, 7693 autosampler, 5975C inert XL MSD; Agilent Technologies, Santa Clara, CA, USA) Transcriptomes: Illumina Hiseq 4000 platform
Data format	Raw, analyzed and formatted
Parameters for data collection	Leaves were obtained from maize plants grown under two conditions, field based OTCs and indoor growth chamber, under either elevated (560 μmol mol^−1^) or ambient CO_2_ (380 μmol mol^−1^). Maize plants were grown under two CO_2_ treatments for 20 days after radicle emergence they were then subjected to a 2 h sudden heat shock stress.
Description of data collection	Following the heat stress, the leaves were immediately immersed into liquid nitrogen for metabolism and transcriptomes.
Data source location	Beltsville Agricultural Research Centre (BARC), United State Department of Agriculture-Agricultural Research Service.
Data accessibility	Data are presented in this article in the form of figures (Fig. 1, Fig. 2, Fig. 3, Fig. 4, Fig. 5) and tables (Table 1, Table 2, Table 3, Table 4, Table 5, Table 6).
Related research article	Li et al., 2019. Roles of heat shock protein and reprogramming of photosynthetic carbon metabolism in thermotolerance under elevated CO_2_ in maize. Environ. Exp. Bot.168. doi.org/10.1016/j.envexpbot.2019.103869

**Table:** 

**Value of the Data** • The experimental data presented herein as well as in Ref. [1] can be used to better understand the response of global gene expression in maize under multiple stress conditions. • The generated datasets specifically provide information on the beneficial effect of elevated CO_2_ on photosynthetic carbon metabolites in response to sudden heat stress treatments. • The expression of heat shock protein in response to CO_2_ treatments can be also learned from this study. • Positive relationship regarding the photosynthetic carbon metabolites between field-based open top chambers (OTCs) and indoor growth chamber was investigated herein. • The data can be used for reference of metabolite quantification and allow other researchers to extend the statistical analysis.

## 1 Data

**Fig. 1:**
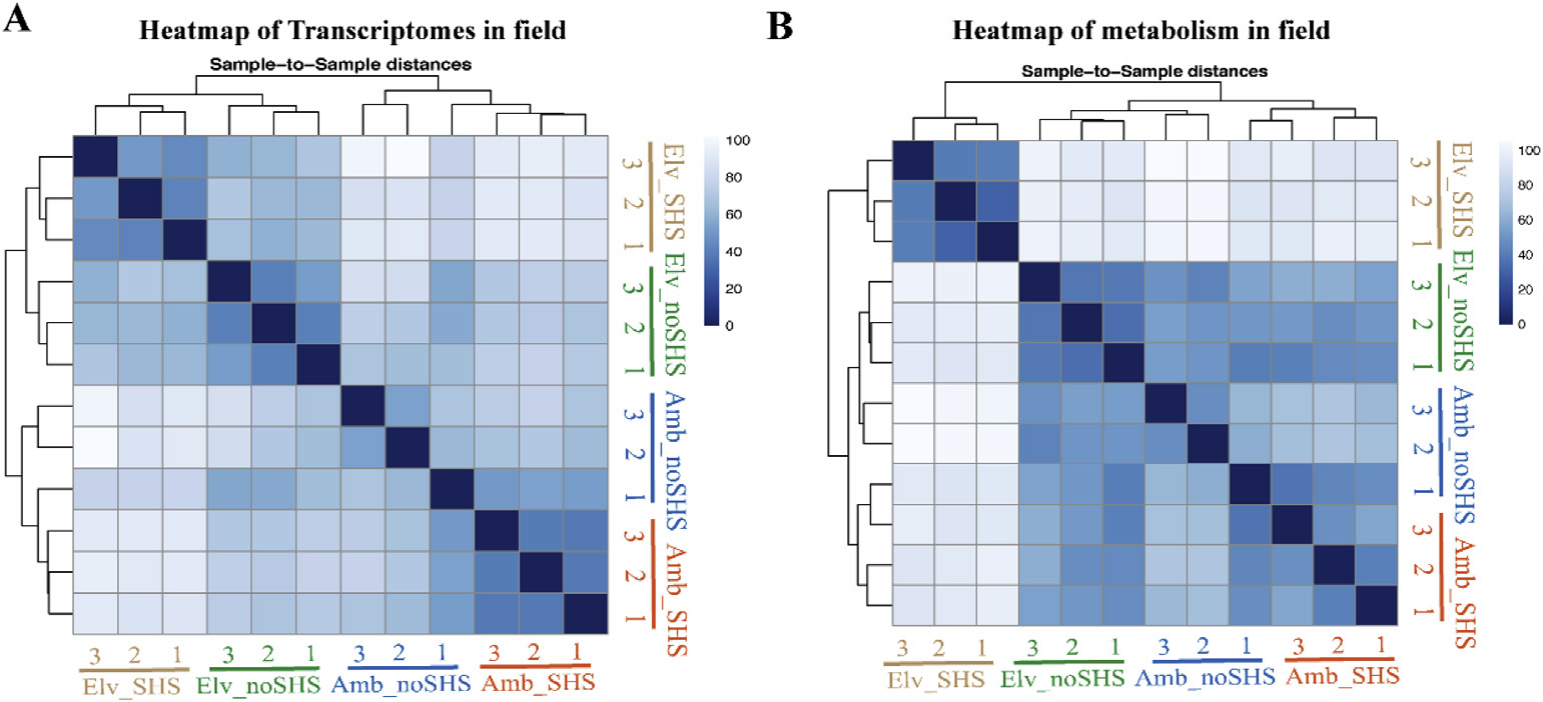
Relatedness of biological samples of maize leaves exposed to combined SHS and elevated CO_2_ grown in field. Heatmap of transcriptomes (A) and metabolism (B) in field. Three biological replicates were performed.

**Fig. 2:**
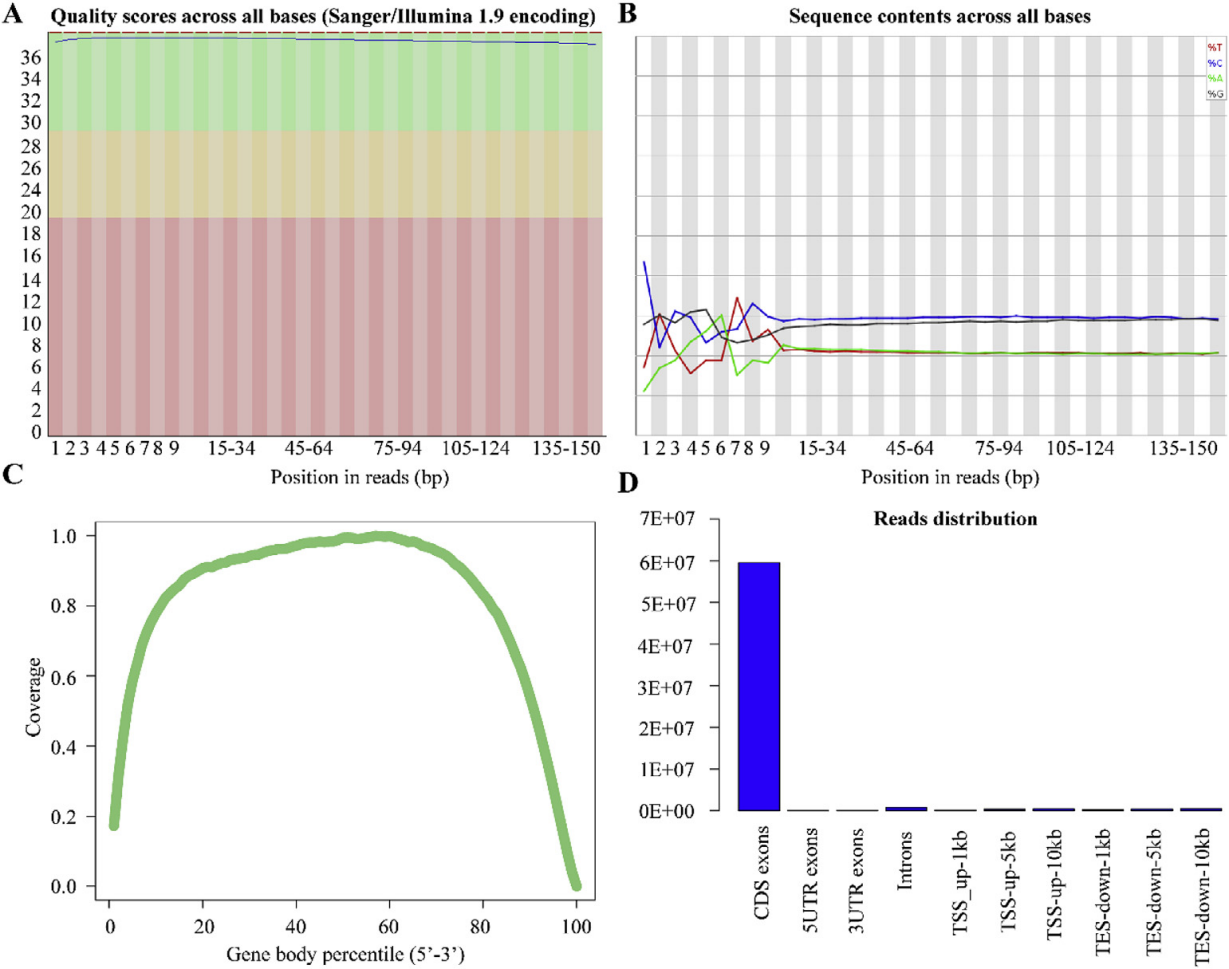
Statistical analysis on quality control of samples for transcriptomes across growth chamber and field. Quality scores (A) and sequence contents (B) across all bases were performed based on transcriptomes analysis. Coverage and distribution of mapped reads across gene body were shown in panels C and D, respectively.

**Fig. 3:**
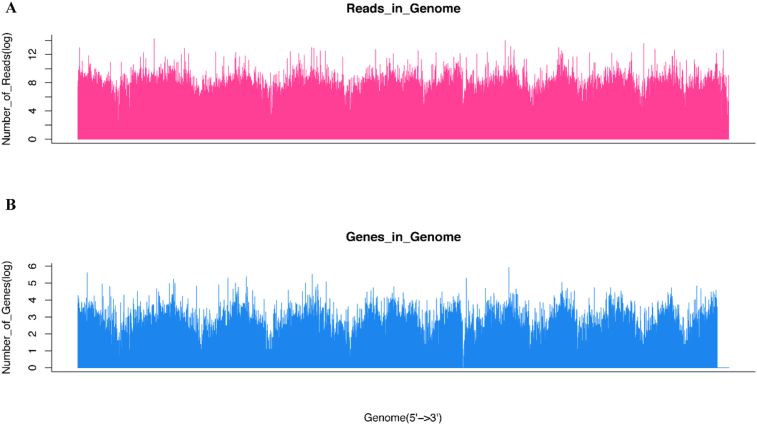
Statically analysis on distribution density of samples for transcriptomes across growth chamber and field trails. Distribution density regarding reads (A) and genes (B) in whole genome.

**Fig. 4:**
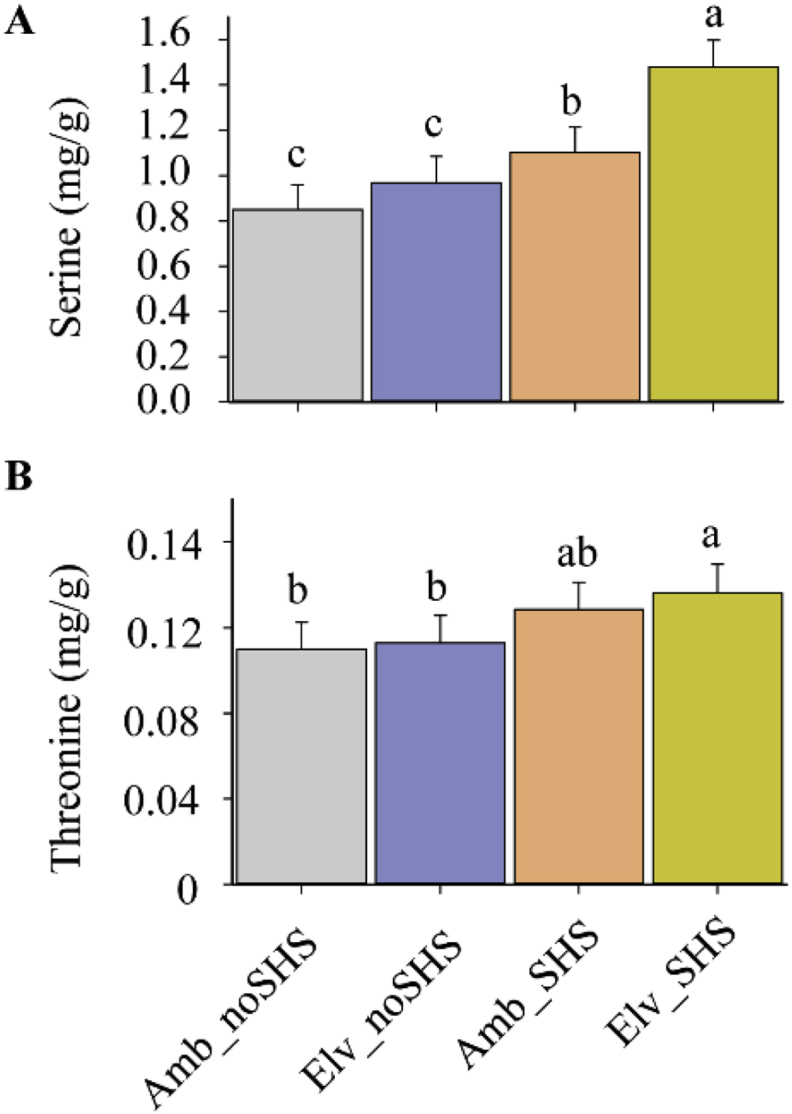
Comparison on metabolites involved in serine and threonine metabolic pathways reprogrammed following combined SHS and elevated CO_2_. Three biological replicates were carried out.

The data collected for SQ maize cultivar exposed to combined effects of elevated CO_2_ and sudden heat stress is presented in five segments of data: A) The relatedness of biological samples in four combination of CO_2_ and SHS regarding to transcriptomes and metabolism in field OTCs conditions as shown in Fig. 1; B) Statistical analysis on sequencing quality across all bases from transcriptomes analysis in field OTCs (Figs. 2 and 3; Table 1); C) GO and KEGG analysis on enriched biological pathway involved in SHS and CO_2_ response (Table 2, Table 3); D) Abundance of heat shock protein based on transcriptomes in different SHS and CO_2_ treatments in growth chamber (Table 4); E) Photosynthetic carbon metabolites and the gene expression of their catalysing enzymes induced by SHS and CO_2_ effects (Fig. 4; Table 5, Table 6). The data included herein are based on the experimental results provided in a previous publication by present authors [1].

**Table 1:** Statistical analysis on numbers of reads for maize leaves subjected to different treatments grown under growth chamber and field.

Sample	Total reads	Total Mapped	Multiple mapped	Uniquely mapped
Amb_noSHS_GR	44,368328	40,863700 (92.10%)	1,916847 (4.32%)	38,946853 (87.78%)
Amb_SHS_GR	47,988,680	44,721,762 (93.19%)	1,845,032 (3.84%)	42,876,730 (89.35%)
Elv_noSHS_GR	45735862	41343632 (90.40%)	2,182589 (4.77%)	39,161043 (85.62%)
Elv_SHS_GR	46,357,682	42,675427 (92.06%)	2,028225 (4.38%)	40,647202 (87.68%)
Amb_noSHS_Field	44,677962	41,109030 (92.01%)	1,968769 (4.41%)	39,140261 (87.61%)
Amb_SHS_Field	46,396662	42,652023 (91.93%)	1,953693 (4.21%)	40,698330 (87.72%)
Elv_noSHS_Field	47,617920	43,657634 (91.68%)	1,874869 (3.94%)	41,782765 (87.75%)
Elv_SHS_Field	47911496	43,937795 (91.71%)	1,901400 (3.97%)	42,036395 (87.74%)

**Table 2:** Gene ontology (GO) analysis on biological pathway enriched from differentially expressed genes induced by SHS with up-regulation of elevated CO_2_.

GO ID	Term	Category	P valule	Enrichment score
GO:0006351	transcription, DNA-templated	biological_process	1.49E-07	1.44015704
GO:0009737	response to abscisic acid	biological_process	9.24E-07	2.43903502
GO:0010,161	red light signaling pathway	biological_process	2.12E-06	19.6231454
GO:0006021	inositol biosynthetic process	biological_process	2.33E-06	10.9017474
GO:0070,413	trehalose metabolism in response to stress	biological_process	4.84E-06	5.98038717
GO:0006952	defense response	biological_process	5.87E-06	1.82692626
GO:0006741	NADP biosynthetic process	biological_process	1.03E-05	15.6985163
GO:0005992	trehalose biosynthetic process	biological_process	1.95E-05	5.10520856
GO:0080,163	regulation of protein serine/threonine phosphatase activity	biological_process	2.65E-05	7.69535114
GO:0010,072	primary shoot apical meristem specification	biological_process	2.65E-05	7.69535114
GO:0005886	plasma membrane	cellular_component	4.63E-05	1.34131818
GO:0070,449	elongin complex	cellular_component	0.00022,912	8.72139796
GO:0005779	integral component of peroxisomal membrane	cellular_component	0.00156,164	5.60661297
GO:0005615	extracellular space	cellular_component	0.00264,417	2.25877933
GO:0005887	integral component of plasma membrane	cellular_component	0.00347,733	1.71231635
GO:0005578	proteinaceous extracellular matrix	cellular_component	0.0044,664	3.60885433
GO:0048,046	apoplast	cellular_component	0.00729,448	1.60063304
GO:0003700	transcription factor activity, sequence-specific DNA binding	molecular_function	1.24E-14	1.95021467
GO:0004512	inositol-3-phosphate synthase activity	molecular_function	8.05E-08	16.3526212
GO:0004760	serine-pyruvate transaminase activity	molecular_function	8.08E-08	20.9313551
GO:0050,281	serine-glyoxylate transaminase activity	molecular_function	8.08E-08	20.9313551
GO:0004445	inositol-polyphosphate 5-phosphatase activity	molecular_function	4.69E-07	17.4427959
GO:0052,658	inositol-1,4,5-trisphosphate 5-phosphatase activity	molecular_function	4.69E-07	17.4427959
GO:0052,659	inositol-1,3,4,5-tetrakisphosphate 5-phosphatase activity	molecular_function	4.69E-07	17.4427959
GO:0043,565	sequence-specific DNA binding	molecular_function	1.39E-06	1.85799281
GO:0016,161	beta-amylase activity	molecular_function	1.62E-06	9.23442136
GO:0003951	NAD^+^ kinase activity	molecular_function	1.03E-05	15.6985163

**Table 3:** Kyoto Encyclopedia of Genes and Genomes (KEGG) analysis on metabolic pathway enriched from differentially expressed genes induced by SHS with up-regulation of elevated CO_2_.

KEGG ID	Term	P value	Enrichment score
path:zma00062	Fatty acid elongation	0.00021,537	5.534060847
path:zma00760	Nicotinate and nicotinamide metabolism	0.00068,782	6.896291209
path:zma00500	Starch and sucrose metabolism	0.0012,358	2.668207908
path:zma02010	ABC transporters	0.00124,559	5.976785714
path:zma00052	Galactose metabolism	0.00127,888	4.038,368,726
path:zma00650	Butanoate metabolism	0.00206,036	5.273634454
path:zma00710	Carbon fixation in photosynthetic organisms	0.00358,693	3.320436508
path:zma00630	Glyoxylate and dicarboxylate metabolism	0.00551,224	3.049380466
path:zma00562	Inositol phosphate metabolism	0.00571,992	2.758516484
path:zma00600	Sphingolipid metabolism	0.01022607	3.448145604
path:zma00250	Alanine, aspartate and glutamate metabolism	0.01244944	2.846088435
path:zma00280	Valine, leucine and isoleucine degradation	0.02105207	2.801618304
path:zma00051	Fructose and mannose metabolism	0.02133673	2.490327381
path:zma00940	Phenylpropanoid biosynthesis	0.02318281	1.854864532
path:zma04146	Peroxisome	0.0239,811	2.230143923
path:zma00564	Glycerophospholipid metabolism	0.02896395	2.01464687
path:zma00270	Cysteine and methionine metabolism	0.03275973	2.075272817
path:zma04016	MAPK signaling pathway - plant	0.03674623	1.83497807
path:zma00030	Pentose phosphate pathway	0.03702845	2.359257519
path:zma00260	Glycine, serine and threonine metabolism	0.05842218	2.037540584

**Table 4:** Transcripts from RNAseq and qPCR results in terms of 17 Heat shock protein gene family in indoor growth chambers. no ch.: means no change.

Maize ID	Gene annotation	Gene abbre.	log2FC (SHS/ck)	Significant	Regulate	Log2FC (eCO_2_/aCO_2_)	Significant	Regulate	Orthologue in Arabidopsis
GRMZM2G458208	cpn1 - chaperonin 1	*Cpn1*	1.9	yes	up	−0.284	no	no ch.	AT3G23990
GRMZM2G416120	cpn2 - chaperonin2	*Cpn2*	0.5356	yes	up	2.4825	yes	up	AT3G23990
GRMZM2G310431	hsp1 - heat shock protein1	*Hsp1*	0.4664	yes	up	3.9482	yes	up	AT3G12580
Zm00001d028555	hsp10 - heat shock protein10	*Hsp10*	0.3535	yes	up	−1.384	no	down	AT1G47890
GRMZM2G306679	hsp11 - heat shock protein11	*Hsp11*	0.4522	yes	up	−0.9482	no	no ch.	AT1G53540
GRMZM2G422240	hsp17.2 - heat shock protein17.2	*Hsp17.2*	0.2553	yes	up	3.4858	yes	up	AT5G12020
GRMZM2G404249	hsp18a - 18 kda heat shock protein18a	*Hsp18a*	0.21,093	yes	up	5.92,874	yes	up	AT5G59720
GRMZM2G034157	hsp18c - heat shock protein18c	*Hsp18c*	0.21,985	yes	up	0.9482	no	no ch.	AT5G12020
GRMZM2G083810	hsp18f - heat shock protein18f	*Hsp18f*	0.2052	yes	up	2.4924	yes	up	AT5G12020
GRMZM2G007729	hsp22 - heat shock protein22	*Hsp22*	0.2132	yes	up	2.94,823	yes	up	AT5G51440
GRMZM2G149647	hsp26 - heat shock protein26	*Hsp26*	0.1942	yes	up	−1.94,824	no	no ch.	AT4G27670
GRMZM6G199466	hsp3 - heat shock protein3	*Hsp3*	0.0942	yes	up	−0.928	no	no ch.	EFH47634.1
GRMZM2G069651	hsp4 - heat shock protein4	*Hsp4*	−0.042	no	no ch.	0.09482	no	no ch.	AT1G53540
GRMZM2G340251	hsp70-4 - heat shock protein70-4	*Hsp70*	0.0486	no	no ch.	0.0838	no	no ch.	AT5G56000
GRMZM2G080724	hsp8 - heat shock protein8	*Hsp8*	0.095	yes	up	1.2948	no	no ch.	AT4G27670
GRMZM2G046382	hsp9 - heat shock protein9	*Hsp9*	0.1821	yes	up	1.94,823	no	no ch.	AT1G47890
GRMZM5G833699	hsp90 - heat shock protein, 90 kDa	*Hsp90*	0.1284	yes	up	0.098,482	no	no ch.	AT5G52640

**Table 5:** Targeted metabolites relevant to metabolic pathways enriched by GO and KEGG analysis with CO_2_ thermal-mitigation effects in indoor growth chambers.

Cluster	#	Metabolites	Amb_noSHS		Elv_noSHS		Amb_SHS		Elv-SHS	
			Mean	S.E.	Mean	S.E.	Mean	S.E.	Mean	S.E.
Carbohydrates	1	starch	9.845^a^	0.041	11.682^a^	0.046	1.517^c^	0.020	3.330^b^	0.027
	2	sucrose	77.723^a^	1.330	66.872^a^	1.624	79.764^a^	1.921	76.602^a^	1.638
	3	trehalose	0.331^a^	0.003	0.437^a^	0.006	0.166^b^	0.004	0.346^a^	0.003
	4	fructose	12.148^b^	0.323	16.621^a^	0.437	5.259^c^	0.349	11.518^b^	0.358
	5	mannose	1.618^a^	0.013	1.422^a^	0.021	0.580^b^	0.012	0.671^b^	0.020
Amino acids	1	valine	0.213^b^	0.021	0.254^b^	0.005	0.894^a^	0.139	1.364^a^	0.136
	2	leucine	0.346^c^	0.035	0.301^c^	0.030	0.765^b^	0.077	1.038^a^	0.104
	3	isoleucine	0.138^c^	0.014	0.158^bc^	0.006	0.183^b^	0.018	0.249^a^	0.025
	4	glycine	1.427^b^	0.019	1.267^b^	0.015	1.934^a^	0.033	2.108^a^	0.031
	5	threonine	2.747^b^	0.315	2.821^b^	0.322	3.209^a^	0.321	3.399^a^	0.340
	6	alanine	2.014^b^	0.295	2.083^b^	0.302	3.010^a^	0.301	3.564^a^	0.319
	7	serine	0.850^b^	0.109	0.967^ab^	0.117	1.102^a^	0.110	1.479^a^	0.118
Organic acids	1	glyoxylate	0.551^c^	0.019	0.548^c^	0.017	1.837^b^	0.007	2.216^a^	0.011
	2	aspartate	7.891^a^	0.037	7.379^a^	0.033	6.289^b^	0.078	6.121^b^	0.071
	3	glutamate	5.232^c^	0.064	3.867^d^	0.052	8.807^a^	0.127	6.604^b^	0.101
	4	pyruvate	0.648^b^	0.017	0.773^b^	0.021	0.106^a^	0.018	0.155^a^	0.018
	5	citrate	1.063^a^	0.016	1.027^a^	0.019	0.105^b^	0.016	0.255^b^	0.017

**Note:** Metabolic responses of maize leaves to CO_2_ and heat stress treatments were presented as: ambient CO_2_ with non-heat stress (Amb_noSHS), elevated CO_2_ with non-heat stress (Elv_noSHS), ambient CO_2_ with heat stress (Amb_SHS), elevated CO_2_ with heat stress (Elv-SHS). One-way *ANOVA* was used to estimate the significant effects of CO_2_ and heat stress on each metabolite in maize leaves, while different alphabet letters represent significant difference at *P* < 0.05.

**Table 6:** FPKMs from RNAseq relating to carbon assimilation metabolic pathways in indoor growth chambers.

Maize ID	Gene name	Abbreviation	Amb_noSHS	Elv_noSHS	Amb_SHS	Elv_SHS	log2FC(SHS/ck)
GRMZM2G069486	β-amylase	*AMY8*	9.088	18.398	2.682	14.324	0.537
GRMZM2G068943	Trahalose 6-phosopate synthase	*TPS*	0.381	0.414	0.147	0.314	0.572
GRMZM6G477257	Phosphoglucose isomerase	*PGI*	12.682	18.701	6.325	17.263	0.711
GRMZM2G129246	Glycolate oxidase	*GO1*	0.402	0.449	0.846	1.170	2.356
GRMZM2G382914	Phosphoglycerate kinase	*PGK2*	0.613	0.759	0.182	0.290	0.340
GRMZM2G438998	Mannose phosphate isomerase	*MPI*	1.550	2.257	0.639	1.801	0.605
GRMZM2G053939	Alanine transaminase	*GPT2*	2.208	2.260	2.130	2.200	0.969
GRMZM2G452630	Serine hydroxymethyltransferase	*SHMT*	1.363	1.197	1.853	1.910	1.477
GRMZM2G473001	PEP kinase	*PEPC*	1.181	1.159	1.110	1.015	0.908
GRMZM2G407044	Acetolactate synthase	*ALS*	0.349	0.312	0.621	0.730	2.059
GRMZM2G094939	Pyruvate dehydrogenase	*PDH*	0.289	0.408	0.275	0.203	0.724
GRMZM2G064023	Citrate synthase	*CS1*	1.353	1.582	0.654	1.365	0.673
GRMZM2G142863	2-oxoglutarte dehydrogenase	*OGDH*	1.015	1.048	0.278	0.097	0.184
GRMZM2G178415	Glutamate dehydrogenase	*GLUD1*	4.893	4.864	7.371	6.991	1.472
GRMZM2G146677	Aspartate transaminase	*AST*	7.736	7.547	6.397	6.566	0.848
GRMZM2G050570	Threonine synthase	*TS2*	0.270	0.250	0.275	0.267	1.043

## 2 Experimental design, materials, and methods

### 2.1 Materials and growth condition

**Fig. 5:**
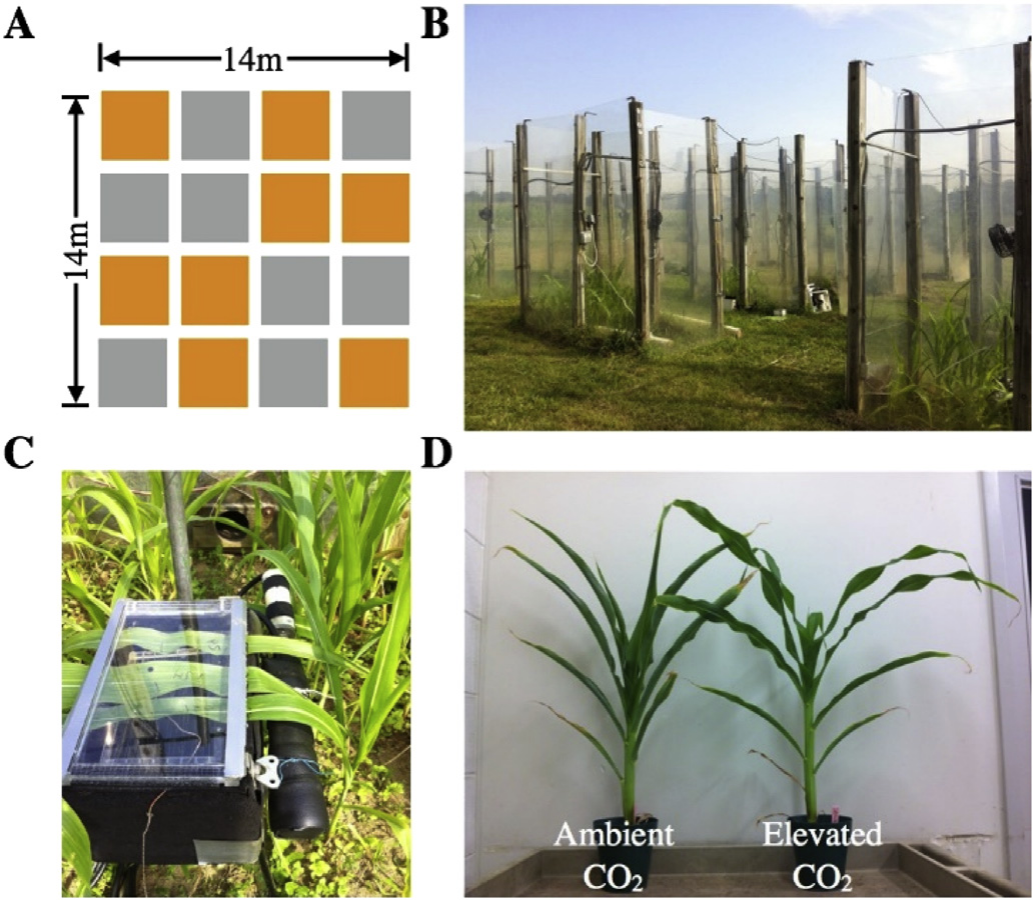
Field experimental design and set-up. (**A**) 4 × 4 randomized block design for field-open top chamber (OTCs) experiments. Ambient and elevated CO_2_ chambers were shown in grey and yellow cells, respectively. (**B**) Image of field OTCs. (**C**) Image of water-jacketed leaf cuvettes. (D) Image of maize grown under ambient (left) and elevated (right) CO_2_ conditions for 20 days.

SQ Corn seeds were supplied by the maize germplasm information resources from the United States of America, USA (GRIN: http://www.ars-grin.gov/). Experiments were conducted in both fields-based open top chambers (OTCs), and indoor conditions. The location of field is at Beltsville Agricultural Research Center (BARC), USDA-ARS (39–00′ N, 76–56′W). The designed 4/4 random blocks for the experiment are as displayed in Fig. 5A. After germination, Corn seedlings were sown in 16 OTCs. The dimension for each OTC is: 2 m long, 2 m width and 2 m height (Fig. 5B). The interval between chambers is uniformly spaced by 2 m, to minimize shading effect. Maize seedlings for 7 days after radicle emergence were transplanted and spaced by 15 cm between each other as well. The soil in each OTC keeps moist by watering once a week. Plants in OTC are exposed to ambient air or ambient air plus 180 ppm CO_2_, as described elsewhere [2].

For indoor chambers, plants were grown under either ambient CO_2_ (380 μmol mol^−1^) or high CO_2_ (560 μmol mol^−1^) concentrations, as described earlier [3]. Day and night temperatures were 29/17 °C, with soil temperature average of 25.7 ± 0.33 °C/14.8 ± 0.41 °C day/night. The light intensity and photoperiod were 1000 μmol m^−2^ s^−1^ and 12/12 h, respectively. Local air humidity was 60% during the day time.

### 2.2 Experimental design

SQ corn variety grown in fields OTCs and growth chambers for 20 days under ambient and high concentrations of CO_2_ as mentioned above. The marked part of the whole intact leaves is placed in a water jacketed leaf chamber (Fig. 5C), with the internal radiator and fan for 2 hours of SHS treatment as described earlier [4]. By circulating heated water from the temperature control tank to the leaf cuvettes (Fig. 5C), the air temperature in the cuvette could increase to approximately 45 °C. Air from the OTCs is constantly flushed through each leaf cuvette. Untreated or heat-treated leaves were immediately stored in liquid nitrogen for transcription and metabolic analysis.

### 2.3 Metabolism measurements

Leaves from six different plants around 20-day old were used (Fig. 5D) for metabolic measurements. ∼30 mg leaf tissue with frozen dried is squashed by adding 3.2 mm ceramic beads and 100 μl fine pomegranate powder in 2.0 mL Eppendorf tube, followed by homogeniztion with a Tissue Lyzer ball mill at 30 cycles s^−1^ as previously described [4]. The squashed samples were subsequently dissolved using 50 μl mixture consisting of 2.5 mM alpha-aminobutyric acid, 2.0 mg ribitol and 1.4 mL cold 70% methanol and vortexed. Then the mixture was incubated in a water bath at 45 °C for 15 min. After centrifugation for 5 min at 12,000 g, super-fluid was gently transferred to a 15 mL fresh conical plastic centrifuge tube. The particles are washed once with 70% methanol, and the supernatants were combined with prevoius step. Finally, the mixed supernatants were air-dried overnight and used for determination of starch as previously described [5]. Organic acids, amino acids and soluble carbohydrates were measured by gas chromatography coupled to mass spectrometry (GC-MS) as described elsewhere [6]. Derived samples are performed by GC-MS equipped with mass selective detection (7890 GC system, 7693 automatic sampler, 5975C idle XL MSD). Total ion chromatograms obtained were quantified using Agilent MSD Chemstation software program. Independent standard curves were prepared for each set of extractions with known mixtures of organic acids, amino acids and soluble carbohydrates. Ribitol added during extraction process as internal standard. Compounds in organic acid fraction: 2-oxoglutaric, quinic acid, adipic acid, shikimate, pyruvate, citrate, aconitate, maleic acid, malate, oxalic acid, malonic acid, glyoxylate, fumarate and succinate. Compounds in soluble carbohydrate fraction were: ribose, fructose, glucose, myo-inositol, sucrose, maltose, mannose, trehalose, raffinose and starch. The compounds present in amino acids fraction: leucine, Isoleucine, alanine, glycine, serine, valine, threonine, proline, putrescine, aspartate, glutamate and phenylalaine. Five biological replicates, with three technique replicates for each biological one, were conducted for metabolic measurements. Values of standard error (SE) were calculated based on data from three technique and five biological replicates. One-way analysis of variance (*ANOVA*) via software SPSS 10.0 (SPSS Inc., USA) was applied to identify significant differences between heat stress and CO_2_ treatments for specific metabolite in SQ maize cultivar leaves.

### 2.4 Transcriptomes measurements

Total RNA was extracted using TRIzol® reagents, following manufacturer's instructions (Invitrogen, Carlsbad, California). Quality and purity of RNA were determined by 1% of agarose gels and nano-drop (IMPLEN, California, USA), respectively. RNA integrity was evaluated via Agilent Bioanalyzer 2100 system (Agilent Technologies, California, USA). The total amount of RNA per sample was normalized to 1.5 μg, which was used as an input for RNA sequencing. Sequencing libraries were generated using NEBNext® UltraTMRNA Library Prep Kit for Illumina® (NEB, USA). Sequencing libraries was featured by Illumina Hiseq 4000 platform with 150bp pair-read was generated [7]. The original read was aligned with B73 reference genome (RefGen_v3), using TopHat2.0.8 and STAR, with a minimum inner length set to 20bp. The gene and heterogeneous are quantified using the GTF annotation file generated by PacBio sequencing. To reduce transcription noise, gene is included only if FPKM value is < 0.01. The value is selected based on the genetic coverage saturation analysis as reported previously [8].
